# Distinguishing the importance between habitat specialization and dispersal limitation on species turnover

**DOI:** 10.1002/ece3.745

**Published:** 2013-08-29

**Authors:** Shixiong Wang, Xiaoan Wang, Hua Guo, Weiyi Fan, Haiying Lv, Renyan Duan

**Affiliations:** 1 College of Life Sciences, Shaanxi Normal UniversityXi'an, 710119, China; 2 School of Life Sciences, Anqing Normal UniversityAnqing, 246011, China

**Keywords:** Loess Plateau, neutral theory, niche assembly, randomization model, variation partitioning

## Abstract

Understanding what governs community assembly and the maintenance of biodiversity is a central issue in ecology, but has been a continuing debate. A key question is the relative importance of habitat specialization (niche assembly) and dispersal limitation (dispersal assembly). In the middle of the Loess Plateau, northwestern China, we examined how species turnover in Liaodong oak (*Quercus wutaishanica*) forests differed between observed and randomized assemblies, and how this difference was affected by habitat specialization and dispersal limitation using variation partitioning. Results showed that expected species turnover based on individual randomization was significantly lower than the observed value (*P* < 0.01). The turnover deviation significantly depended on the environmental and geographical distances (*P* < 0.05). Environmental and spatial variables significantly explained approximately 40% of the species composition variation at all the three layers (*P* < 0.05). However, their contributions varied among forest layers; the herb and shrub layers were dominated by environmental factors, whereas the canopy layer was dominated by spatial factors. Our results underscore the importance of synthetic models that integrate effects of both dispersal and niche assembly for understanding the community assembly. However, habitat specialization (niche assembly) may not always be the dominant process in community assembly, even under harsh environments. Community assembly may be in a trait-dependent manner (e.g., forest layers in this study). Thus, taking more species traits into account would strengthen our confidence in the inferred assembly mechanisms.

## Introduction

Elucidating the underlying processes that shape community patterns through space and time is a central issue in ecology (Gilbert and Lechowicz 2004). Niche assembly and dispersal assembly have frequently been cited as primary determinants of species distribution (Hubbell 2001; Tuomisto et al. 2003). Under niche assembly, the assembly of communities is controlled by the match between species niches and local environmental conditions (i.e., habitat specialization or environmental filtering), and sites with similar ecological conditions should harbor similar species assemblages (Tuomisto et al. 2003; Jones et al. 2006). Under dispersal assembly, dispersal limitation governs patterns of distribution among species whose ecological abilities are predicted to be largely equivalent (Hubbell 2001), and sites should harbor increasingly dissimilar species assemblages with increasing between-site spatial distances (Nekola and White 1999). Due to the polarity, the overall importance of these two processes has been a continuing debate (Hubbell 2001; Tuomisto et al. 2003; Karst et al. 2005). Recent evidences suggested that niche and dispersal processes may not be mutually exclusive, but with varying relative importance across different spatial scales, regions, and plant groups (Gravel et al. 2006; Normand et al. 2006; Legendre et al. 2009). For example, habitat specialization has a higher explanatory power than dispersal limitation in temperate forests, whereas dispersal limitation is the main driver of plant species dissimilarity in the tropical forests (Myers et al. 2013). Clearly, more efforts are needed to make generalizations about relative contributions of habitat specialization and dispersal limitation to species distribution patterns (Lin et al. 2013).

Floristic patterns and species distributions have been studied using various beta diversity proxies, and have generated a growing confusion about the appropriate metric for measurement (Jurasinski et al. 2009; Tuomisto 2010a). Species turnover quantifies the changes of species composition among compositional units (Tuomisto 2010a,b), and is an important tool for understanding processes that drive diversity patterns (Freestone and Inouye 2006; Laliberté et al. 2009; Kraft et al. 2011). For example, variation partitioning and Mantel tests are often used to estimate the relative importance of niche and dispersal processes through partitioning the variation in community composition (species turnover) between environmental and posteriori-selected spatial factors/distances (Tuomisto et al. 2003; Jones et al. 2006; Laliberté et al. 2009; Legendre et al. 2009).

The Loess Plateau in northwestern China is well known for its deep loess. However, recently, it has attracted wide attention due to its severe soil erosion as a result of human settlement and other activities (Zhou et al. 2013). Land resources in this area are seriously disturbed due to the intensive soil erosion. For instance, one ton loess soil is estimated to contain 0.8–1.5 kg of total nitrogen, 1.5 kg of total phosphorus, and 20 kg of total potassium (Cai 2001; Zhou et al. 2013). Therefore, the niche-determined process (habitat specialization) is widely considered as the dominant process in this area due to its harsh environments, whereas dispersal limitation is often neglected. However, recent studies have extensively proved that dispersal limitation is also a key process for temperate forests (Gilbert and Lechowicz 2004; Laliberté et al. 2009; Myers et al. 2013). More importantly, the Loess Plateau is characterized by obvious habitat fragmentation due to human activities (Jiang et al. 2003; Wang 2006), which may strengthen dispersal limitation. Therefore, disentangling their effects between dispersal limitation and environmental filtering on plant community assembly in this area is of extreme importance for strengthening biodiversity conservation and vegetation restoration.

Thus, the major objective of this study was to ascertain the relative influences of the habitat specialization and dispersal limitation on the assembly of plant community in the Loess Plateau. Specifically, we used Liaodong oak (*Quercus wutaishanica*) forests as a model forest, which is the potential natural vegetation. To test for nonrandom species turnover, we generated null distributions using a randomization approach that reshuffled the observations according to standard methods (Crist et al. 2003; Freestone and Inouye 2006). Then, we identified nonrandom ecological processes that disproportionately differentiate regional diversity by analyzing the compositional relationships between environmental factors and spatial variables using Mantel tests and variation partitioning (Legendre et al. 2009; Lindo and Winchester 2009; Lin et al. 2013). We predicted (1) that community patterns should be nonrandom, and randomized community species turnover should be smaller than the observed value due to some combination of dispersal limitation and habitat specialization (Crist et al. 2003; Myers et al. 2013); and (2) that their relative contributions of dispersal limitation and habitat specialization should vary among forest layers, as these two processes heavily depend on species traits (e.g., height and growth form) (Flinn et al. 2010; Kristiansen et al. 2012). However, habitat specialization should be the dominant process in this area due to its harsh environments (Nakashizuka 2001).

## Material and Methods

### Study site

This study was conducted in the Mts. Ziwuling, in the middle of the Loess Plateau, northwestern China. The region has a temperate continental monsoon climate with a mean annual temperature of 7°C (ranging between −7°C in January and 18.3°C in July) and annual precipitation equaling 580 mm (primarily occurring between June and September). The altitude ranges from 1200 m to 1700 m. Some primary forests have been replaced by Chinese pine (*Pinus tabulaeformis*) plantations and naturally regenerated forests. Liaodong oak forest is the potential natural vegetation in this area and patchily distributed among landscapes.

### Field sampling

**Figure 1:**
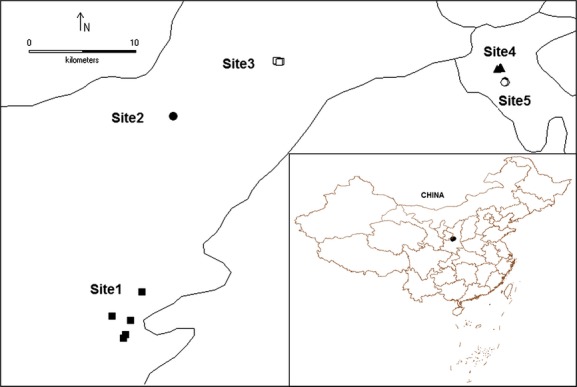
Schematic map of the study area showing the locations of sampling sites.

Five typical Liaodong oak forest sites were selected over a 30-km range in the Mts. Ziwuling (Fig. 1), and surveyed using a hierarchical nested sampling design within the five sites. Vascular plants in each site were divided into three layers (i.e., herb layer, shrub layer, and canopy layer) according to their growth form and height. Five 20 m × 20 m plots were established at each site. Three nested subplots with different sizes were established in each quadrant of the plots to identify the canopy layer (10 m × 10 m), the shrub layer (4 m × 4 m), and the herb species (1 m × 1 m). A total of 100 subplots (5 sites × 5 plots × 4 subplots) were established at each layer. Data from the four subplots were pooled at the plot level (*n* = 25). The canopy layer (height >3 m) comprises all tree species and some tall shrub species. The shrub layer (height between 1 m and 3 m) comprises shrub species and saplings of some tree species. The herb layer (height <1 m) mainly comprises herb species and seedlings of some shrub and tree species.

Several environmental variables were measured in each plot (Table S1). Slope aspect (Aspect) is a circular variable; sin (Aspect) and cos (Aspect) were computed in order to use slope aspect in linear models (Legendre et al. 2009). The geographical coordinate and elevation of each plot were determined using a handheld global positioning system (GPS) receiver. Slope degree was measured with a clinometer. The depths of leaf-litter and humus were also measured. At each plot, soil salinity was determined with a TDR Hydra Probe System (Stevens Water Monitoring Systems, Inc., Beaverton, OR). We pooled five soil samples from each plot and soil nutrients were analyzed (Liang et al. 2010). Soil pH was measured electrometrically (10 g soil in 20 mL 0.01 mol/L CaCl_2_). Soil organic matter content (SOM) was determined by K_2_Cr_2_O_7_ oxidation and FeSO_4_ titration. The available nitrogen (N) was determined by the continuous alkali hydrolyzed reduction diffusing method. The available phosphorus (P) was determined by the Mo–Sb anticolorimetric method. The available potassium (K) was determined by flame photometric determination.

### Null model and species turnover

We hypothesized that habitat specialization coupled with some degree of dispersal limitation best explains current plant distributions. We compared observations against a null model that assumes no limitation on dispersal (i.e., everything can be everywhere) and no habitat specialization (i.e., the environment does not favor the growth of specific plants). Our null model is similar to Hubbell's neutral theory in that it lacks environmental forcing; however, it is distinct in that it assumes no limits on species dispersal (Sul et al. 2013).

First of all, we defined the species pool as the total number of species and the total abundance of each species observed across all plots within a region. Next, we measured observed species turnover as the dissimilarity between each pair of plots within a region using an abundance-based (Bray–Curtis) metric (Myers et al. 2013). Then, we applied the null model to simulate species assemblages in each plot by randomly sampling individuals from the regional species pool while preserving the relative abundance of each species in the regional pool and the total number of individuals in each plot (Crist et al. 2003; Kraft et al. 2011). From 1000 iterations of the null model, we calculated a standardized effective size (turnover deviation) as the difference between the observed and mean expected species turnover, divided by the standard deviation of expected values. Then, Student's *t*-test was used to determine whether mean turnover deviation differed significantly from zero (at the significance level of α = 0.05). A turnover deviation of zero indicates that observed species turnover does not differ significantly from random sampling, a positive turnover deviation indicates higher species turnover than expected by chance and a negative turnover deviation indicates lower species turnover than expected by chance.

### Variation partitioning

To disentangle habitat specialization, dispersal limitation, or a combination of both processes, two complementary approaches (i.e., Mantel tests and canonical variation partitioning) were employed.

First, Mantel tests and partial Mantel tests were performed to test whether the magnitude of turnover deviation depended on the geographical distance (GeoD) (as a proxy for dispersal limitation) and environmental differences (as a proxy for habitat specialization) as possible drivers of dissimilarity in plant species composition (Kristiansen et al. 2012). The datasets were identical to that for the canonical variation partitioning. Both geographical and environmental distances (EnvDs) were based on Euclidean distance.

Second, canonical variation partitioning was used to identify the relative contribution between habitat specialization and dispersal limitation through partitioning the variation in community composition between environmental and posteriori-selected spatial factors (Gilbert and Lechowicz 2004; Legendre et al. 2009). Canonical variation partitioning was performed by redundancy analysis (RDA) and partial redundancy analysis (pRDA). We removed variables that were highly correlated with other variables (Pearson's correlation coefficient *r* > 0.80) to account for collinearity among environmental variables, yielding a total of 11 environmental variables (Table S1). The environmental factors were as follows: soil salt, P, K, SOM, pH, litter depth, humus depth, elevation, slope degree, sin (Aspect), and cos (Aspect). The 14 spatial variables were obtained using the principal coordinates of neighboring matrices (PCNM) analysis (Borcard and Legendre 2002; Dray et al. 2006). For a complete description of the method, see Dray et al. (2006).

Environmental data were standardized (i.e., *z*-transformed) prior to the analysis. Species abundance data were “Hellinger” transformed (Legendre and Gallagher 2001). For each analysis, we used forward selection (Monte Carlo permutation, *n* = 999) to retain only the significant environmental and spatial variables in the final model (*P* < 0.05) using “packfor” package (Dray et al. 2009). The total variation in the dependent species matrices was broken down into the following components: [E|S] = the fraction of species variation that can be explained by environmental factors independent of any spatial structure, [E∩S] = variation explained by spatially structured environmental factors, [S|E] = the fraction of the variation that can be explained by spatial factors independent of any environmental factors (as a proxy for dispersal limitation), and the unexplained variation 1 − [E + S] (Gilbert and Lechowicz 2004; Lindo and Winchester 2009). The *R*^*2*^ values were adjusted to account for the number of sampling sites and explanatory variables, as unadjusted *R*^*2*^ values are biased (Peres-Neto et al. 2006). The canonical variation partitioning, and tests of significance of the fractions were computed using the “vegan” library (Oksanen et al. 2010) of the R statistical language (R Development Core Team 2008). PCNM variables were created with the program “SpaceMaker” (Borcard and Legendre 2004).

## Results

**Figure 2:**
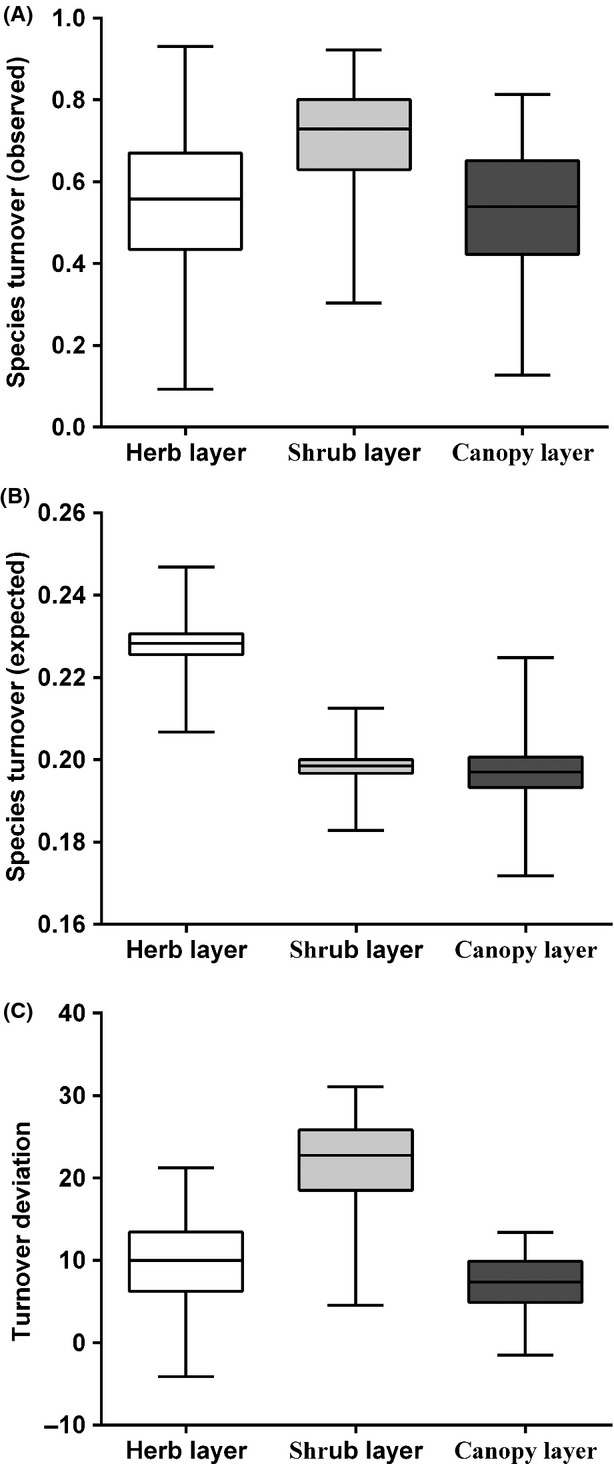
Species turnover for the three layers: (A) observed species turnover (Bray–Curtis dissimilarity), (B) expected species turnover from a null model based on random sampling from the regional species pool, and (C) turnover deviation, a standardized effective size of species turnover that controls for sampling from the regional species pool. Boxes represent the median and 25th/75th percentile, and upper and lower edges represent the maxim and minim values. Note that turnover deviations are strongly positive, indicating higher species turnover than expected by chance.

In total, 80 herb layer species, 102 shrub layer species, and 34 canopy layer species were recorded at the five sites. Observed species turnover was higher at shrub layer than at herb and canopy layers (Fig. 2). The expected species turnover based on individual randomization was significantly lower than the observed value (*P* < 0.01). The turnover deviation was roughly similar among the three layers (slightly higher at shrub layer) (Fig. 2). The turnover deviations were strongly positive at all three layers, reflecting strong intraspecific aggregation of most species.

Turnover deviation was significantly correlated with both geographical and EnvDs (*P <* 0.05), and the correlation coefficients varied among layers (Table 1). The herb and shrub layers had a larger correlation coefficient with EnvD, even after controlling for the effect of GeoD in a partial Mantel test (Table 1). In contrast, the canopy layer community dissimilarity displayed larger correlation coefficients with GeoD, even after controlling for the effect of EnvD (Table 1).

**Table 1:** Mantel test and partial Mantel test correlations for turnover deviation, geographical distance (GeoD), and environmental distance (EnvD) for the three layer species

Matrices used	Herb layer		Shrub layer		Canopy layer	
		
	*R*	*P*	*R*	*P*	*R*	*P*
EnvD	0.52	0.001	0.45	0.001	0.17	0.016
EnvD|GeoD	0.39	0.003	0.34	0.001	0.12	0.069
GeoD	0.38	0.002	0.32	0.001	0.26	0.005
GeoD|EnvD	0.09	0.043	0.05	0.161	0.23	0.005

EnvD|GeoD, turnover deviation with environmental distance, controlling for geographical distance; GeoD|EnvD, turnover deviation correlations with geographical distance, controlling for environmental distance.

**Figure 3:**
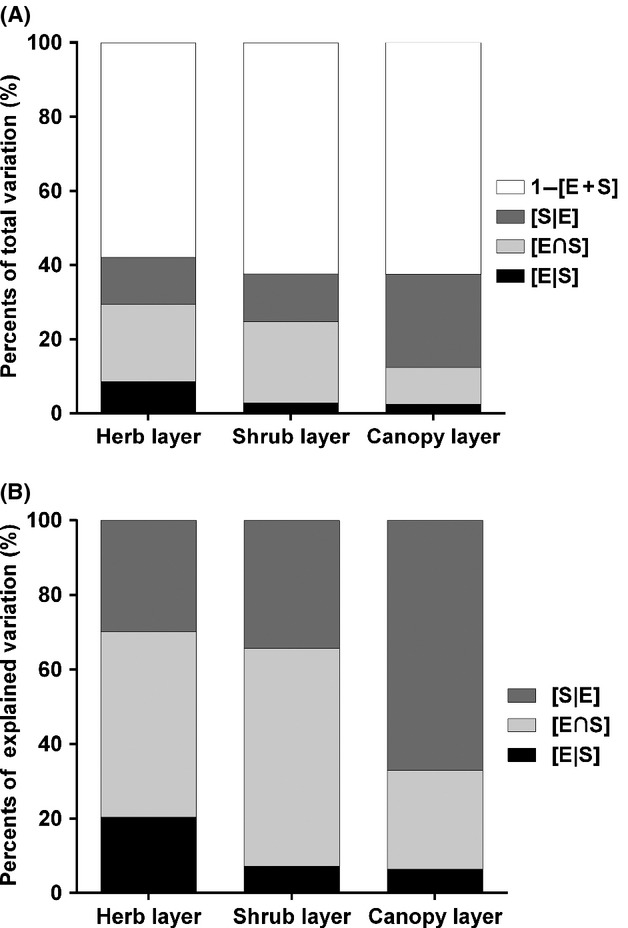
Variation partitioning for different layer species: (A) percents of total variation and (B) percents of explained variation. Fractions [E]−[S] (adjusted *R*^*2*^ statistics, 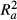): [E|S] = the fraction of species variation that can be explained by environmental factors independent of any spatial structure, [S|E] = the fraction of the variation that can be explained by spatial factors independent of any environmental factors, [E∩S] = variation explained by spatially structured environments, and 1 − [E + S] = the unexplained variation.

Environmental and spatial variables significantly explained approximately 40% of the species composition variation at all three layers (*P* < 0.01) (Fig. 3). Pure spatial variables [S|E] significantly explained a larger variation in species composition (*P* < 0.05), especially for the canopy layer. However, pure environmental variables [E|S] only significantly explained the smaller proportion of species composition variation for the herb layer. Overall, the understory layers (i.e., the herb and shrub layers in this study) dominated by environmental contributions [E] ([E] = [E|S] + [E∩S]), and the joint environmental and spatial variables [E∩S] accounted for a greater share than either set of predictors independently. In contrast, the canopy layer was dominated by space (percentages of explained variation >50%; Fig. 3). The significant environmental variables differed among forest layers, and were as follows: elevation, K, pH, and SOM for the herb layer species; elevation, SOM, pH for the shrub layer species; SOM and slope aspect for the canopy layer species (Table 2).

**Table 2:** Explanatory variables selected by the forward selective procedure in the RDA (*P* < 0.05)

		Variable	*AdjR*^*2*^*Cum*	*F*	*P*
Environment	Herb layer	Elevation	0.11	4.03	0.001
		Available potassium (K)	0.21	3.72	0.001
		pH	0.26	2.53	0.003
		Soil organic matter (SOM)	0.29	2.09	0.003
	Shrub layer	Elevation	0.09	3.52	0.001
		Soil organic matter (SOM)	0.20	4.07	0.001
		pH	0.25	2.32	0.004
	Canopy layer	Soil organic matter (SOM)	0.06	2.56	0.017
		cos (Aspect)	0.12	2.63	0.019
Space	Herb layer	PCNM5	0.11	3.95	0.001
		PCNM2	0.22	4.21	0.001
		PCNM1	0.28	2.94	0.001
		PCNM3	0.33	2.66	0.001
	Shrub layer	PCNM5	0.11	4.08	0.001
		PCNM2	0.22	3.97	0.001
		PCNM1	0.29	3.37	0.001
		PCNM3	0.35	2.85	0.001
	Canopy layer	PCNM1	0.20	6.89	0.001
		PCNM2	0.25	2.46	0.018
		PCNM5	0.30	2.63	0.009
		PCNM3	0.35	2.74	0.007

PCNM, Principal coordinates of neighbor matrices. *AdjR*^*2*^*Cum*, adjusted cumulative square of the sum of all canonical eigenvalues (expressing explained variance). *F, F-*test statistic. *P-*value refers to the significance of the variable (Monte Carlo permutation test). RDA, redundancy analysis.

## Discussion

Our findings supported that habitat specialization and dispersal limitation are both necessary to understand community assembly in the Loess Plateau forests. The magnitude of the turnover deviation significantly depended on the EnvDs and GeoDs. Moreover, the significant contribution of environmental and pure spatial variables suggested that habitat specialization and dispersal limitation were two important processes that determined community patterns (Fig. 3). Similar results were also obtained using the distance-based method, i.e., multiple regressions on distance matrices (Fig. S1). Therefore, our results are consistent with the widely held viewpoint that niche processes and neutral assembly (e.g., dispersal limitation) are not mutually exclusive, but may work together to determine species diversity and species coexistence (Gilbert and Lechowicz 2004; Freestone and Inouye 2006; Chase 2007; Legendre et al. 2009), further supporting the continuum hypothesis (Gravel et al. 2006).

As expected, the environment contributed significantly for all three layers, and was the dominant process for the understory layers (i.e., the herb and shrub layers in this study). Among the examined environmental variables, SOM was the common important environmental factor for the three layers. In addition, elevation, slope aspect and other soil nutrients were also significant factors (Table 2). Studies in Amazonia (Tuomisto et al. 2003) and Indonesia (Paoli et al. 2006) also indicated that soil nutrients and topography are important factors affecting species turnover, determining species composition probably through processes such as resource competition (Stevens and Carson 2002) and recruitment limitation (Grubb 1977). It was a salient feature that the spatially structured component ([E∩S]) explained such a large proportion of variation in community composition (Fig. 3), which can be explained by the fact that the dominant contributions of primarily environmental factors (e.g., soil nutrients) were spatially structured. This was confirmed by the Mantel test, which showed that environmental variables were significantly related to GeoD (*P* < 0.05).

Contrary to our prediction, however, the more significant contributions of pure spatial indicated that dispersal limitation was also an important process which shaped community patterns, even as a dominant process for the canopy layer (Fig. 3). Although the fraction explained by pure space is usually linked to dispersal processes (Gilbert and Lechowicz 2004), other spatially structured environmental factors that were not included in the analysis may also contribute (Legendre et al. 2009), leading to an overestimation of the purely spatial fraction (Diniz-Filho et al. 2012). In this study, we are most interested in the spatial structure which arises from dispersal characteristics of species (i.e., dispersal limitations); the arrangement and/or connectivity of suitable habitats would support our hypotheses (Lindo and Winchester 2009), as dispersal limitation may heavily depend on the degree of habitat connectivity (e.g., fragmentation). In fact, fragmentation and patchy distribution are obvious characteristics of forests in the Loess Plateau due to human activities (Jiang et al. 2003; Wang 2006). Fragmentation can promote species turnover through the creation of barriers for dispersal, the modification in patch size and shape, and the generation of variation in microclimatic effects, all of which were unfavorable for the arrival and establishment of species (Honnay et al. 1999; Bascompte and Rodríguez 2001). For instance, fruit production and disperser abundance are often lower in fragments, which causes reductions in seedling density due to seed limitation (da Silva and Tabarelli 2000; Bruna 2002). Our inference was further confirmed by the significant association between GeoD and turnover deviation (Table 1), whereas GeoD was always used as a proxy for dispersal limitation (Kristiansen et al. 2012; Tuomisto et al. 2012). Moreover, the importance of dispersal limitation has also been extensively proved in the temperate forests using different methods, such as seed addition experiments (Tuomisto et al. 2003; Bustamante-Sánchez and Armesto 2012; Myers et al. 2013). In conclusion, identifying the relative influence of dispersal limitation is of particular importance for our understanding the community assembly, for which dispersal may be the first step for community assembly (Egler 1954).

Overall, the understory layers were mainly controlled by habitat specialization, whereas canopy layer was mainly dominated by dispersal limitations. The results were also consistent with species traits prediction. Taller canopy layer species are always less environmentally specialized than understory species due to their large sizes and strong root systems (Ricklefs and Latham 1992). So patterns of canopy layer will demonstrate a significant spatial signature, i.e., dispersal limitation. In contrast, the understory layer displayed an opposite signature, i.e., habitat specialization. However, such comparisons should be performed with caution, as environmental and spatial predictors are afflicted by different sources of error (Smith and Lundholm 2010; Kristiansen et al. 2012). For instance, the effect of environmental factors is affected by the quality of measurements, as well as the range of measured variables (Jones et al. 2008). In the case of dispersal limitation, the pure spatial contribution can be also related to unmeasured environmental factors that are themselves spatially structured. Despite these limitations of our approach and that of previous studies (Jones et al. 2008; Laliberté et al. 2009; Legendre et al. 2009), the difference in magnitude between pure spatial [S|E] and environmental effects [E] is so large that it is likely robust. Therefore, our study contributes to our understanding of the relative influence of environmental versus spatial drivers of species turnover in temperate forests in the Loess Plateau.

Although habitat specialization and dispersal limitation are main mechanisms that may structure biodiversity (Gilbert and Lechowicz 2004; Freestone and Inouye 2006; Legendre et al. 2009), it should be noted that about 60% of the variation was unexplained (1 − [E + S]; Fig. 3). One possible explanation is that other nonspatially structured biological or environmental factors that are not measured in the field may ultimately be responsible for such partitioning (Legendre et al. 2009). Another plausible explanation is the stochastic processes, which have theoretical connection to the neutral theory of macroecology assuming that the dynamics of populations are primarily driven by ecological drift and dispersal, with or without limitation, and are habitat-independent (Legendre et al. 2009). For instance, communities showed significantly higher similarity among ponds after experiencing drought; this had likely resulted from niche assembly which filtered out some less competitive species (i.e., those unable to tolerate such environmental harshness) from the regional pool (Chase 2007). However, there was considerable site-to-site variation in pond community composition in the absence of drought, which had likely resulted from a combination of stochastic ecological drift and priority effects.

## Conclusion

This study provides mechanistic insights into the assembly and maintenance of biodiversity in a community characterized by harsh environments. First, our results underscore the importance of synthetic models that integrate effects of both dispersal and niche assembly for understanding the community assembly. Second, our results indicate that dispersal limitation is important for understanding the forest community assembly, even under harsh environmental conditions, and thus will contribute to the implementation of ecologically based management actions to preserve the remaining forest fragments. Third, consistent with previous findings on trait-related process relationships, our observations suggest that the processes of dispersal limitation and habitat specialization along with environmental gradients have differential importance to plants with different traits (e.g., growth form and height of species in this study), even to those occurring within the same communities. To fully understand community patterns, dividing community into different functional groups based on more species traits (e.g., dispersal ability and habitat affinity) and identifying mechanisms that link functional groups with ecological processes should be the next important task.

## Supporting Information

**Figure S1.** Variation partitioning based on distance matrices for different layer species: (A) percents of total variation and (B) percents of explained variation.

**Table S1.** Profiles for the study sites in Mts. Ziwuling.
