# Supplementary material for: Distinguishing the importance between habitat specialization and dispersal limitation on species turnover
Source: Ecol Evol. 2013 Aug 29;3(10):3545–53. doi: 10.1002/ece3.745 (PMC3797498; doi:10.1002/ece3.745)
Supplement: Supplementary file 1 [file ece30003-3545-SD1.doc]

**Table S1. Profiles for the study sites in Mts. Ziwuling.**

|  | **Site 1**  (mean ± SD) | **Site 2**  (mean ± SD) | **Site 3**  (mean ± SD) | **Site 4**  (mean ± SD) | **Site 5**  (mean ± SD) | **All sites**  (range) |
| --- | --- | --- | --- | --- | --- | --- |
| Herb layer species richness (No./plot) | 11.2 ± 3.8 | 7.2 ± 2.8 | 10.2 ± 3.1 | 17.0 ± 5.9 | 18.0 ± 3.2 | 5-24 |
| Shrub layer species richness (No./plot) | 25.8 ± 1.6 | 24.8 ± 1.9 | 24.0 ± 2.2 | 26.2 ± 4.8 | 19.2 ± 6.3 | 11-33 |
| Canopy layer species richness (No./plot) | 7.4 ± 1.5 | 8.0 ± 0.7 | 7.6 ± 1.9 | 8.4 ± 1.7 | 5.2 ± 1.5 | 3-11 |
| Available nitrogen (N; mg kg^-1^) | 17.8 ± 9.2 | 15.0 ± 4.7 | 115.2 ± 14.5 | 61.8 ± 61.4 | 124.6 ± 49.3 | 8.8-195.8 |
| Available phosphorus (P; mg kg^-1^) | 9.5 ± 3.7 | 13.0 ± 4.8 | 8.8 ± 2.9 | 15.5 ± 8.1 | 20.1 ± 1.6 | 5.0-25.5 |
| Available potassium (K; mg kg^-1^) | 76.5 ± 46.8 | 40.5 ± 16.0 | 134.8 ± 26.4 | 89.5 ± 59.0 | 225.7 ± 29.2 | 25.0-251.8 |
| Soil organic matter (SOM; g kg^-1^) | 6.0 ± 2.2 | 6.1 ± 1.3 | 28.1 ± 3.4 | 15.4 ± 11.2 | 36.2 ± 13.8 | 2.9-55.5 |
| Soil pH | 7.5 ± 0.2 | 7.1 ± 0.1 | 7.4 ± 0.1 | 7.6 ± 0.3 | 8.1 ± 0.3 | 7.0-8.5 |
| Soil salt | 0.2 ± 0.03 | 0.2 ±0.01 | 0.3 ± 0.05 | 0.2 ± 0.05 | 0.2 ± 0.03 | 0.17-0.34 |
| Litter depth (cm) | 2.6 ± 0.3 | 2.4 ± 0.5 | 2.6 ± 0.6 | 2.5 ± 0.5 | 2.8 ± 0.8 | 1.7-4.0 |
| Humus depth (cm) | 2.8 ± 0.6 | 2.9 ± 0.7 | 2.5 ± 0.8 | 2.6 ± 0.7 | 3.8 ± 0.8 | 1.0-5.0 |
| Elevation (m) | 1471 ± 20 | 1537 ± 21 | 1469 ± 11 | 1498 ± 41 | 1711 ± 49 | 1450-1753 |
| Slope degree (°) | 31.6 ± 9.2 | 21.0 ± 4.5 | 23.8 ± 9.3 | 20.2 ± 2.8 | 27.6 ± 6.8 | 10.0-40.0 |
| Slope aspect (°) | 301.8 ± 13.5 | 45.0 ± 13.7 | 210.4 ± 57.6 | 223.0 ± 59.8 | 109.0±131.1 | 15.0-340.0 |


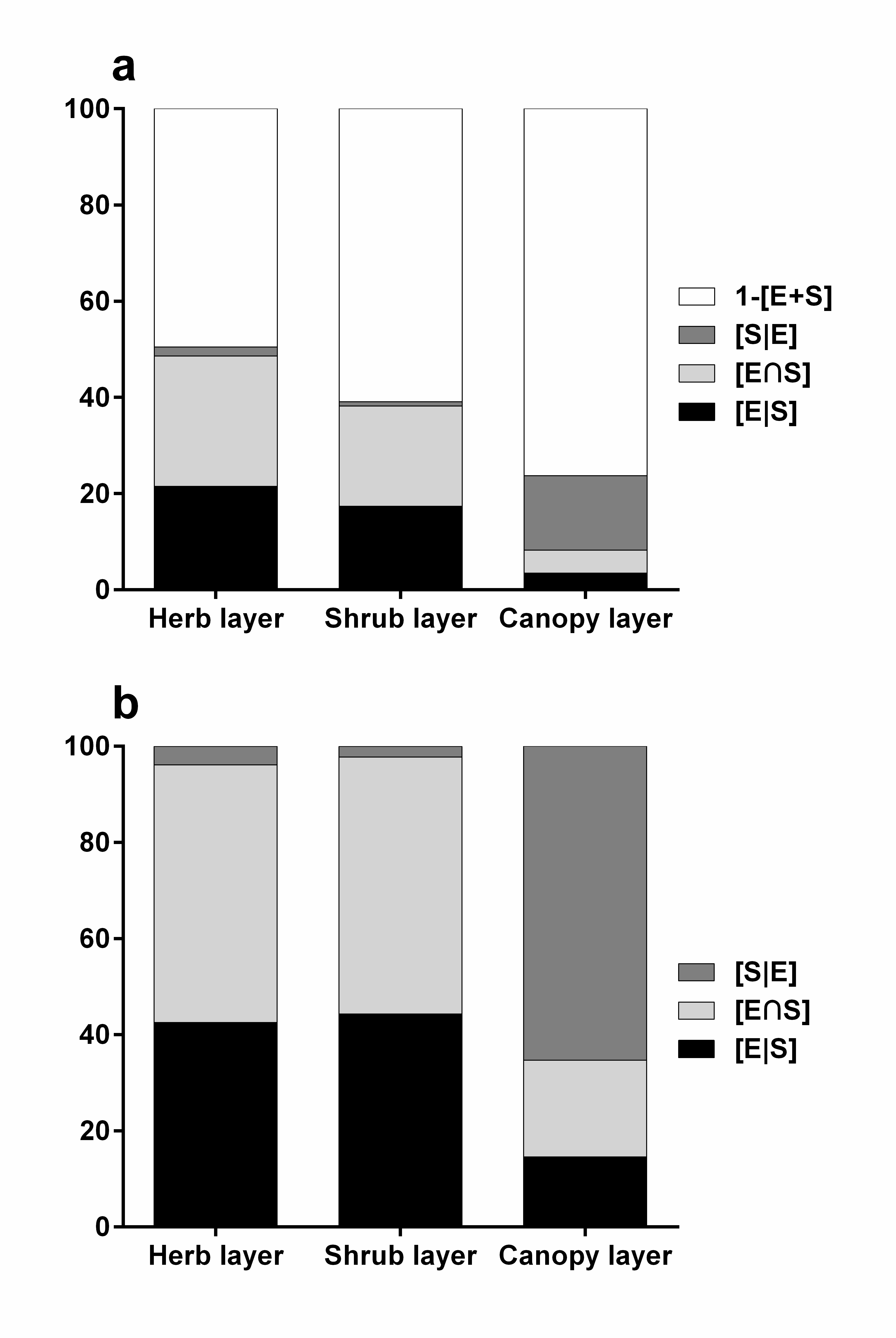


**Figure S1.** Variation partitioning based on distance matrices for different layer species: (a) percents of total variation and (b) percents of explained variation. Fractions [E]-[S] (adjusted *R^2^* statistics,$R_{a}^{2}$): [E|S] = the fraction of beta diversity that can be explained by environmental dissimilarity alone, [S|E] = the fraction of the beta diversity that can be explained by geographical distance alone, [E∩S] = beta diversity explained by spatially structured environmental dissimilarity, and 1-[E+S] = the unexplained variation.
